# Supplementary material for: Intrinsic Disorder in BAP1 and Its Association with Uveal Melanoma
Source: Genes (Basel). 2022 Sep 22;13(10):1703. doi: 10.3390/genes13101703 (PMC9601668; doi:10.3390/genes13101703)
Supplement: Supplementary file 1 [file genes-13-01703-s001.zip › genes-1889968-supplementary.pdf]

genes MDPI

Figure S1. BAP1's amino acid sequence. Shown in FASTA format, the protein sequence represents BAP1 canonical form. The protein contains 729 residues.

>sp|Q92560|BAP1_HUMAN Ubiquitin carboxyl-terminal hydrolase BAP1 OS=Homo sapiens OX=9606 GN=BAP1 PE=1 SV=2
MNKGWLELESDPGLFTLLVEDFGVKGVQVEEIYDLQSKCQGPVYGFIFLFKWIEERRSRRKVSTLVDDTSVIDDDIVN
NMFFAHQLIPNSCATHALLSVLLNCSSVDLGPTLSRMKDFTKGFSPESKGYAIGNAPELAKAHNSHARPEPRHLPEK
QNGLSAVRTMEAFHFVSYVPITGRLFELDGLKVYPIDHGPWGEDEEWTDKARRVIMERIGLATAGEPYHDIRFNLM
AVVPDRRIKYEARLHVLKVNRQTVLEALQQLIRVTQPELIQTHKSQESQLPEESKSASNKSPLVLEANRAPAASEGN
HTDGAEEAAGSCAQAPSHSPPNKPKLVVKPPGSSLNGVHPNPTPIVQRLPAFLDNHNYAKSPMQEEEDLAAGVGR
SRVPVRPPQQYSDDEDDYEDDEEDDVQNTNSALRYKGKGTGKPGALSGSADGQLSVLQPNTINVLAEKLKESQKDL
SIPLSIKTSSGAGSPAVAVPTHSQPSPTPSNESTDTASEIGSAFNSPLRSPIRSANPTRPSSPVTSHISKVLFGEDDSLLRVD
CIRYNRAVRDLGPVISTGLLHLAEDGVLSPLALTEGGKGSSPSIRPIQGSQGSSSPVEKEVVEATDSREKTGMVRPGEPL
SGEKYSPKELLALLKCVEAEIANYEACLKEEVEKRKKFKIDDQRRTHNYDEFICTFISMLAQEGMLANLVEQNISVRR
RQGVSIGRLHKQRKPDRRKRSRPYKAKRQ

Table S1. UM-related mutations in human *BAP1* gene and local disorder predisposition at sites of mutations in BAP1 protein. *Table starts on next page*.

**ID**	**DNA change** [a]		**Protein change** [a]	**Predicted effect on protein**	**Predicted pathogenic effect**[a]	**Local intrinsic disorder predisposition**[a]	**Reference**
UM 44	exon 1	c.3G>A	p.Met1Ile	misense: start site lost	LP	0.8174	[42]
UM 9	exon 2	c.40_52del13	p.Leu14SerfsTer54	truncating	P	0.1864	[42]
UM 1136	exon 2	c.58_59insTG	p.Glu20ValfsTer53	truncating	P	0.1198	[42]
UM 584	exon 3	c.79delG	p.Val27CysfsTer45	truncating	P	0.2189	[42]
UM 17	exon 3	c.82C>T	p.Gln28Ter	truncating	P	0.2405	[42]
UM 1207	exon 3	c.91_93delGAG	p.Glu31del	in-frame deletion	LP	0.2474	[42]
NB 071M	exon 3	c.271C>T	p.Gln36Ter	truncating		0.2827	[21]

ID	DNA change [a]		Protein change [a]	Predicted effect on protein	Predicted pathogenic effect[a]	Local intrinsic disorder predisposition[a]	Reference
UM 75	exon 4	c.125_145del21	p.Pro42_Phe48del	in-frame deletion	LP	0.051±0.036[c]	[42]
MM 120	exon 4	c.259delC	p.Phe48fsTer22	truncating		0.0205	[21]
UM 88	exon 4	c.145delC	p.Leu49CysfsTer23	truncating	P	0.0297	[42]
MM 144	exon 4	delG	p.Phe50LeufsTer22	truncating		0.0449	[41]
UM 877	exon 4	c.165_180del16	p.Arg57SerfsTer10	truncating	P	0.4857	[42]
UM 13	exon 4	c.178C>T	p.Arg60Ter	truncating	P	0.5870	[42]
UM_62	exon 4	c.202_227del26	p.Asp68CysfsTer3	truncating	P	0.4115	[42]
UMM 004	exon 4	T>C	p.Asp68Gly	missense		0.4115	[41]
UM 119	exon 4	c.253C>T	p.Gln85Ter	truncating	P	0.1426	[42]
UM 106	exon 4	c.254A>C	p.Gln85Pro	missense	P	0.1426	[42]
UMM 006	exon 5	delTGTGAGCCAGGATGAAGGCA CTGCAGCCTACCTCAGGGCTGA AACCCTTGGTGAAGTCCTTCATG CGACTCAGGGTGGGTCCCAGGTC CACGCTGCTGCAGTTCAGGAGCA CGCTCAGCAAGGCATGAGTTGCA CAAGAGTTGGGTATCAG	p.Leu86_Glu125del	in-frame deletion		0.34±0.17	[41]
UM 1126	exon 5	c.295_312del18	p.Val99_Ser104del	in-frame deletion	LP	0.187±0.033	[42]
MM 129	exon 5	A>C	p.Leu101Arg	missense		0.16395	[41]
MM 090	exon 5	c.467_487del21	p.Asp117fs48	truncating		0.1491	[21]

ID	DNA change [a]		Protein change [a]	Predicted effect on protein	Predicted pathogenic effect[a]	Local intrinsic disorder predisposition[a]	Reference
MM 121	exon 6	c.497G>C	p.Glu128Arg	missense	LP	0.6465	[21]
UM 56	exon 6	c.422A>G	p.His141Arg	missense	LP	0.7665	[42]
MM 173	exon 7	C>A	p.Arg146Met	missense		0.7776	[41]
UM 58	exon 7	c.458_459delCT	p.Pro153ArgfsTer7	truncating	P	0.6711	[42]
NB 191	exon 7	c.610-634del25	p.Met165fsTer12	truncating		0.2419	[21]
UM 114	exon 7	c.497_509del13	p.Glu166ValfsTer17	truncating	P	0.2958	[42]
UM 51	exon 7	c.506A>C	p.His169Pro	missense	P	0.1251	[42]
MM 125	exon 7	c.622C>G	p.His169Gln	missense		0.1251	[21]
NB 200	exon 7	c.631C>G	p.Ser172Arg	missense		0.0900	[21]
UM 1118	exon 7	c.524C>G	p.Pro175Arg	missense	LP	0.0867	[42]
UMM 010	exon 8	delC	p.Leu186Ter	truncating		0.1772	[41]
UM 60	exon 8	c.588G>A	p.Trp196Ter	truncating	P	0.4656	[42]
UM _1334	exon 8	c.619delC	p.Arg207GlyfsTer24	truncating	P	0.2719	[42]
UM 35	exon 9	c.723T>A	p.Tyr241Ter	truncating	P	0.2819	[42]
MM 060	exon 9	c.872C>T	p.Gln253Ter	truncating		0.2764	[21]
UM 107	exon 9	c.781C>T	p.Gln261Ter	truncating	P	0.4608	[42]
UMM 009	exon 9	delA	p.Leu262Argfs*2	truncating		0.5003	[41]

**ID**	**DNA change [a]**		**Protein change [a]**	**Predicted effect on protein**	**Predicted pathogenic effect[a]**	**Local intrinsic disorder predisposition[a]**	**Reference**
MM 066	exon 10	c.960_968del9	p.Glu284-Ser285del	in-frame deletion		0.9585±0.0005	[21]
MM 175	exon 10	delAGCACCAGCGGGGACTTGTTG	p.Ser289ArgfsTer41	truncating		0.9591	[41]
MM 161	exon 10	delCT	p.Arg300GlyfsTer6	truncating		0.9591	[41]
UM 46	exon 10	c.904_905insT	p.Pro302LeufsTer5	truncating	P	0.9596	[42]
MM 070	exon 11	c.1083-1093delCCCCATCCCAC	p.Gln322fsTer100	truncating		0.9535	[21]
UM 1029	exon 12	c.1134_1143del10_insAA	p.Ala379ArgfsTer16	truncating	P	0.7391	[42]
UM 115	exon 12	c.1153C>T	p.Arg385Ter	truncating	P	0.8348	[42]
UM 55	exon 12	c.1175_1182delAGCAGTAC	p.Gln392LeufsTer3	truncating	P	0.9109	[42]
UM 863	exon 12	c.1192G>T	p.Glu398Ter	truncating	P	0.9463	[42]
UMM 007	exon 12	A>T	p.Tyr401Ter	truncating		0.9478	[41]
UM 950	exon 12	c.1203dupT	p.Glu402Ter	truncating	P	0.9457	[42]
UM 69	exon 12	c.1217_1220delAGGA	p.Glu406ValfsTer23	truncating	P	0.9264	[42]
UM 1333	exon 13	c.1695dupT	p.Glu566Ter	truncating	P	0.3981	[42]
MM 110	exon 13	c.182901833delCCCCT	p.Ser571fsTer25	truncating		0.4695	[21]
UM 48	exon 13	c.1729G>C	p.Glu577Gln	missense	P	0.6967	[42]

**ID**	**DNA change** [a]		**Protein change** [a]	**Predicted effect on protein**	**Predicted pathogenic effect**[a]	**Local intrinsic disorder predisposition**[a]	**Reference**
UMM 002	exon 14	delGGCTGCTGGACCCCTGGCTGC CTTGGATTGGTCTGATGGA	p.Ser585Glnfs*19	frame-shift deletion		0.8770	[41]
MM 004	exon 14	T>A	p.Gln590Leu	missense		0.9358	[41]
UM 61	exon 14	c.1881C>G	p.Tyr627Ter	truncating	P	0.7660	[42]
UM 74	exon 14	c.1882_1885delTCAC	p.Ser628ProfsTer8	truncating	P	0.7533	[42]
UM 708	intron 14-3'UTR	c.1890+38_2573del	p.Glu631Ter	truncating (3 exon deletion)	P	0.6899	[42]
NB 185	exon 15	c.2006-2017del12	p.Glu631_Ala634del	in-frame deletion		0.619±0.054	[21]
MM 046	exon 15	c.2026_2928delGTG	p.Lys637_Cys638delinsN	in-frame deletion		0.561±0.003	[21]
UM 1113	exon 15	c.1926_1951del 26	p.Ile643GlyfsTer12	truncating	P	0.4603	[42]
UM 104	exon 15	c.1932_1948deldel17	p.Asn645GlnfsTer13	truncating	P	0.4055	[42]
UM 804	exon 16	c.1986_1989delTGAT	p.Ile662MetfsTer29	truncating	P	0.6474	[42]
NN 128	exon 16	c.211202129delGAAGGACCC	p.Arg666_His669del	in-frame deletion		0.399±0.063	[21]
UM 108	exon 16	c.2015A>G	p.Asp672Gly	missense	LP	0.1914	[42]
MM 152M	exon 17	c.2195_2220del26	p.Glu693fsTer12	truncating		0.3648	[21]

[a] The DNA and protein changes are sourced from a previous studies (cited in last column).
[b] Local intrinsic disorder predisposition was evaluated by PONDR® VSL2 [49, 50].
[b] In cases of the in-frame deletion mutations removing more than one residues, an average disorder score was calculated for the entire deleted region.
